# PpoR is a conserved unpaired LuxR solo of *Pseudomonas putida *which binds *N*-acyl homoserine lactones

**DOI:** 10.1186/1471-2180-9-125

**Published:** 2009-06-17

**Authors:** Sujatha Subramoni, Vittorio Venturi

**Affiliations:** 1 Bacteriology Group, International Centre for Genetic Engineering & Biotechnology, Trieste, Italy

## Abstract

**Background:**

Only a small number of *Pseudomonas putida *strains possess the typical *N*-acyl homoserine lactone quorum sensing system (AHL QS) that consists of a modular LuxR family protein and its cognate LuxI homolog that produces the AHL signal. Moreover, AHL QS systems in *P. putida *strains are diverse in the type of AHLs they produce and the phenotypes that they regulate.

**Results:**

We identified an unpaired LuxR solo (QS *luxR *homolog that occurs without the corresponding *luxI *homolog), which is highly conserved in both the AHL producing and non-AHL producing *P. putida *strains that we analyzed. In this study we report the cloning and functional characterization of this unpaired LuxR homolog designated PpoR. An AHL binding assay showed that PpoR protein binds to 3-oxo-C6-HSL. Studies using a *ppoR *promoter-*lacZ *reporter fusion revealed that it exhibits stringent growth phase dependent expression. Functional interaction of PpoR with the endogenous complete AHL QS systems of *P. putida *WCS358 (PpuI/R system) and PpoR was also investigated. Microarray analysis of *P. putida *WCS358 wild type and a PpoR over-expressing strain revealed several putative target genes that may be directly or indirectly regulated by PpoR.

**Conclusion:**

Our results indicate that PpoR in *P. putida *strains may have a conserved role in detecting an AHL signal, either self or foreign, and regulating specific target genes.

## Background

Most bacteria have a regulatory system, known as quorum sensing (QS), to modulate gene expression as a function of their cell density (for reviews see [1,2]). It usually works via the production of a signaling molecule that reaches a threshold concentration at high cell density allowing its detection by the bacterial population and resulting in the modulation of target gene expression. In gram negative, *N*-acyl homoserine lactone signaling molecules (AHLs) are thus far the most common signal molecules produced. A typical AHL QS system involves two major components: an AHL synthase gene (belonging to the LuxI protein family) and a modular transcriptional response-regulator (belonging to the LuxR protein family) which detects and responds to the AHL concentration [3].

AHL QS thus far is exclusively found in proteobacteria; 68 of 265 sequenced proteobacterial genomes possess at least one *luxI/R *family pair [4]. Interestingly, 90 genomes contained at least one *luxR *gene having the modular characteristics of the QS-family of regulators; however it was not associated with a cognate *luxI*-family gene. Of these, 45 genomes harbor at least one complete AHL QS system in addition to one or more *luxR *gene/s. These unpaired LuxR family proteins were firstly designated orphans [5] and recently they have been proposed to be renamed as LuxR 'solos' [6]; a few of these LuxR solos are beginning to be studied. ExpR of *Sinorhizobium meliloti*, BisR of *Rhizobium leguminosarum *bv. *viciae *and QscR of *Pseudomonas aeruginosa*, are LuxR solo proteins in AHL producing bacteria which have been well characterized and shown to be integrated with the resident complete AHL QS regulatory networks [7-10]. Only two solo LuxR homologs in non-AHL producing bacteria have thus far been investigated in some detail. One is called SdiA which is present in the *Salmonella enterica *and *Escherichia coli *and shown to be able to bind and detect AHLs produced by other bacteria. The other one is from plant pathogenic *Xanthomonas *spp. and in two *Xanthomonas *species it is involved in regulating virulence factors upon binding an unknown plant produced low molecular weight compound which is not an AHL [11-13]. This indicates that certain quorum sensing related LuxR family proteins are able to be involved in inter-kingdom signaling by detecting non-AHL compounds produced by eukaryotes.

*Pseudomonas putida *strains are mainly studied either for their ability to establish beneficial association with plants or due to their versatile catabolic potential. Previous studies have indicated that the majority of soil-borne or plant-associated *P. putida *strains do not produce AHLs; apparently only about one third of strains belonging to these species have a complete AHL QS system [14,15]. Furthermore, the type and role played by these AHL QS systems varies and is highly unpredictable [16]. *P*. *putida *strains appear to be rather unique in displaying such variation and lack of conservation in their AHL QS systems. In this study we report however that a LuxR solo is very well conserved in all *P. putida *strains we tested. This protein, which we designated PpoR, was shown to be able bind to AHLs, was not involved in rhizosphere colonization and was shown to be involved in the regulation of several loci. In addition its gene is stringently growth-phase regulated. The presence and sequence similarity of PpoR and its orthologs in all *P. putida *strains indicates that this protein might play a conserved role associated with the detection and response to bacterial endogenous and/or exogenous signaling compounds.

## Results and Discussion

### PpoR, an unpaired LuxR homolog protein is highly conserved in *Pseudomonas putida*

**Figure 1:**
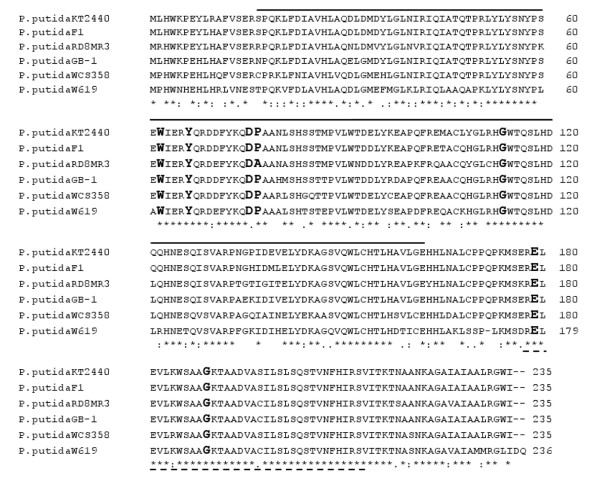
**Alignment showing similarity of deduced sequence of PpoR to its orthologs**. Multiple sequence alignment was performed using the ClustalW2 program (Thompson et al. 1994). The protein sequences used for the alignment are as follows; *P. putida *KT2440 (AAN70220.1), *P*. *putida *F1 (ABQ80629.1), *P. putida *RD8MR3 (this study; accession number FM992078), *P*. *putida *GB-1 (ABZ00528.1), *P. putida *WCS358 (this study; accession number FM992077) and *P*. *putida *W619 (ACA71296.1). The amino acids that are conserved in QS LuxR family proteins are indicated in bold [3]. In the alignment, all identical amino acids (*), similar amino acids (:) and completely different amino acids (.) at a particular position are indicated. Also indicated are the regions of the protein sequence of PpoR of *P. putida *KT2440 that constitutes the AHL binding domain (bold line from 17 to 162 amino acids; PFAM 03472) and the DNA binding domain (dashed line from 176 to 213 amino acids; PFAM 00196).

The model *P. putida *KT2440 has not been reported to possess an AHL QS system and its genome sequence does not encode for a LuxI homolog. As we were interested in studying solo QS LuxR homolog proteins in *P. putida*, the genome sequence of *P. putida *KT2440 (AE015451) was examined for the presence of such proteins that typically contain an *N*-terminal AHL binding domain (PFAM 03472) and a C-terminal helix-turn-helix DNA binding domain (PFAM 00196). A single ORF, PP_4647 of 705 bp was identified encoding a protein of 235 amino acids and named as PpoR (Pseudomonas putida orphan regulator). A BLAST search revealed high similarity to several other *P. putida *strains whose genome sequences, either complete or partial are available in the NCBI database. PpoR exhibits similarity to orthologs from *P*. *putida *F1 (ABQ80629.1; 97%), *P*. *putida *GB-1 (ABZ00528.1; 95%), *P*. *putida *W619 (ACA71296.1; 84%) as well as to its homolog from *P*. *entomophila *L48 (CAK17431; 75%). We were also interested to know if *ppoR *is present in two other *P. putida *strains; namely *P. putida *WCS358 and *P. putida *RD8MR3; these two *P. putida *strains also possess a complete AHL QS system, hence they are able to produce and respond to AHLs [16,17]. It was established that they possess a PpoR ortholog as we have cloned and sequenced *ppoR *from both strains (see Methods; Figure 1). Importantly, all these orthologs along with PpoR of *P. putida *KT2440 retain those five amino acids in their AHL-binding domain that are invariant in this family of proteins (Figure 1; [3]). These observations indicate that PpoR is highly conserved as it is present in all *P. putida *strains that we examined, suggesting that it might be part of the core genome of *P. putida*. On the other hand, approximately only one-third of *P. putida *strains possess a complete AHL QS; in addition, the type and role of these systems is not conserved [16]. Homologs of PpoR are also present in several groups of other bacteria which belong to γ-proteobacteria, β-proteobacteria as well as enterobacteria, all of which show a high similarity of around 50% at amino acid level, indicating conservation across species (data not shown).

### PpoR binds to AHL molecules

**Figure 2:**
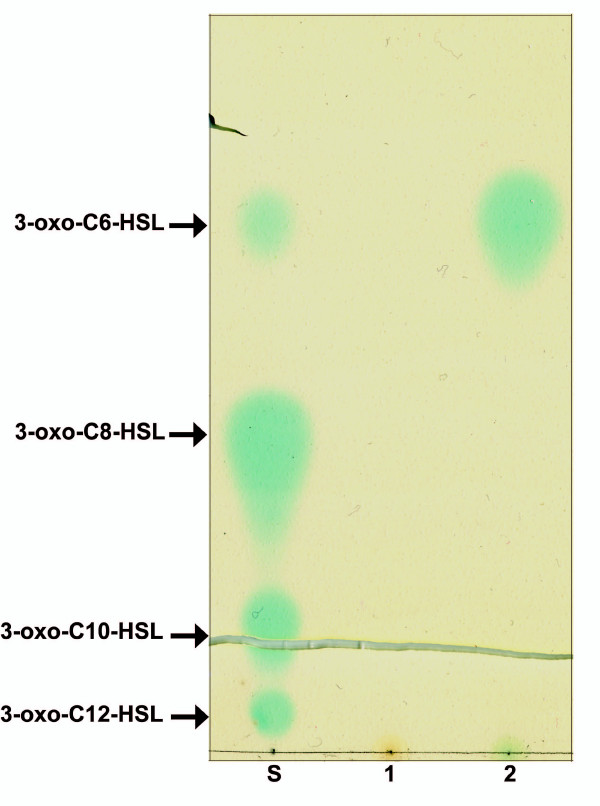
**PpoR binds 3-oxo-C6-HSL**. *E. coli *M15 (pRep4) containing either pQEPpoR or pQE30 were grown in LB in the presence of various AHLs (1 μM) added separately and protein expression induced with IPTG (1 μM). After 3.5 hours of growth post induction, AHLs were extracted from the cell pellets and visualized by TLC overlaid with *A. tumefaciens *NTL4 (pZLR4). The standards used are synthetic AHLs. In the figure, the lanes are marked as follows; S – AHL standards, 1 – AHL extracted from *E. coli*/pQE30 cell pellets grown with 3-oxo-C6-HSL supplementation and 2 – AHL extracted from *E. coli*/pQEPpoR cell pellets grown with 3-oxo-C6-HSL supplementation.

**Figure 3:**
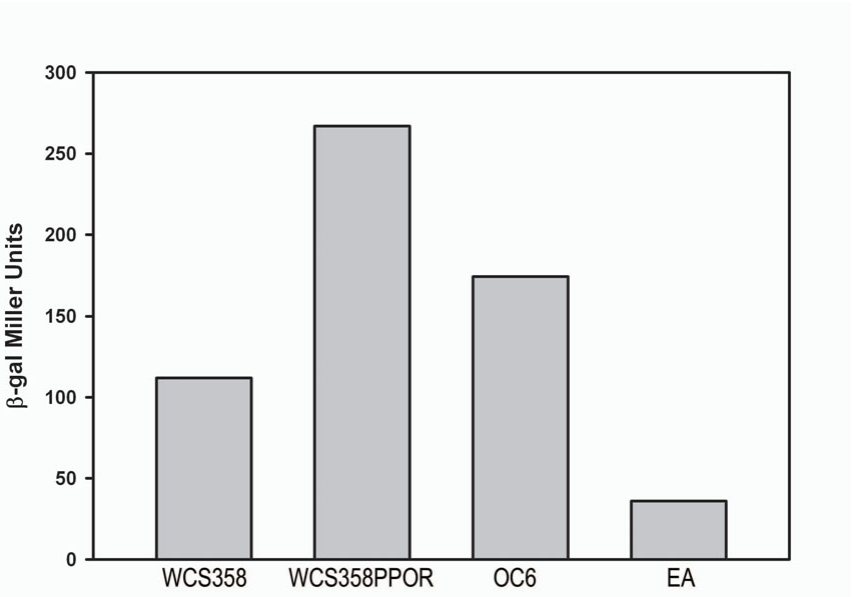
**3-oxo-C6-HSL measurement produced by *P. putida *WCS358 and by the *ppoR *mutant WCS358PPOR**. AHLs were extracted from spent supernatants and levels were measured using a biosensor as described by Steindler et al., [15]. AHL levels were measured using a volume of extract corresponding to an amount of 5 × 10^8 ^cfu as described in the Methods section. 3-oxo-C6-HSL level is proportional to β-galactosidase activity (Miller Units); β-gal refers to β-galactosidase; OC6 refers to 0.05 μM of synthetic 3-oxo-C6-HSL used for the experiment and EA refers to ethyl acetate added as control in the experiment. β-galactosidase activity (Miller Units) represented as bars were obtained from one such experiment; similar values were obtained from additional experiments carried out with AHLs extracted independently from *P. putida *WCS358 and WCS358PPOR strains.

The presence of conserved amino acids in the AHL binding domain of PpoR of *P. putida *KT2440 indicated a possible binding to one or more AHLs. In order to identify if and which AHLs may bind PpoR, an AHL-binding assay was performed. *E. coli *strains that expressed PpoR protein or contained vector alone were grown in the presence of a set of externally supplemented AHLs (unsubstituted, oxo as well hydroxy AHLs) and any AHL that may bind to PpoR was visualized after purification via organic extraction, TLC and overlay with an AHL biosensor/indicator strain (as described in Methods). Purification of AHLs from *E. coli *over-expressing PpoR resulted in detection of 3-oxo-C6-HSL while *E. coli *cells which contained only the vector control, did not show any AHL (Figure 2). These results strongly indicate that PpoR most probably binds to 3-oxo-C6-HSL. Additionally, PpoR also exhibited probable binding to 3-oxo-C8-HSL and 3-oxo-C10-HSL, but to a lower extent at the concentrations of AHLs used in our experiment (data not shown). All the other AHLs tested in our assay could not be detected by TLC meaning over-expression of PpoR did not result in their purification. This could mean that they most probably do not bind to these AHLs or the binding is much lower than the sensitivity of this assay. It was concluded that PpoR of *P. putida *KT2440 and most probably other *P. putida *strains lacking a complete AHL QS system could be sensing and responding to AHL signals produced by neighboring bacteria. PpoR may also recognize endogenous AHL signals if the *P. putida *strain is able to produce AHLs. Interestingly, the few *P. putida *strains reported to possess a complete AHL QS system produce 3-oxo-C6-HSL [16-18], which as shown in this study could bind PpoR. In order to verify that *P. putida *WCS358 produced biologically active concentrations of 3-oxo-C6-HSL, we quantified these AHL levels in the wild type and in the *ppoR *mutant strains. As can be seen from Figure 3, strain WCS358 produced biologically active concentrations of AHLs and interestingly the *ppoR *mutant produced higher quantities. The reason for this is currently unknown, it cannot be excluded that lack of PpoR in the cells could result in higher quantities of free AHLs since as shown above, PpoR can bind and titrate away 3-oxo-C6-HSL.

### PpoR interacts with the endogenous AHL QS system of *P. putida *WCS358

To study the role of PpoR in *P. putida *WCS358 and *P. putida *RD8MR3 which also have a resident AHL QS system, knock-out mutants in *ppoR *were generated in both these strains (Table 1; Methods). The AHL production profile of the *ppoR *mutant was similar to the one of the WT with only a reproducible slight increase in the intensity of the signal for WCS358PPOR and lower intensity than wild type for RD8MR3PPOR (data not shown). Quantification of the amount of signal produced by the *ppoR *mutant (using two biosensors specifically detecting AHLs produced by WCS358 and one for the AHLs produced by strain RD8MR3), showed a similar trend of the *ppoR *mutants producing slightly more AHLs for WCS358 and slightly less AHLs for RD8MR3 (Figure 3 for data on quantification of 3-oxo-C6-HSL; for quantification of 3-oxo-C12-HSL data not shown).

**Table 1:** Bacterial strains and plasmids used in this study

Bacteria, plasmids or primers	Characteristics	Reference or source
Pseudomonas putida		
P. putida RD8MR3	Wild type; isolated from rhizosphere of rice roots	[24]
P. putida RD8MR3PPRI		[16]
P. putida RD8MR3PPRR	pprR::Km of P. putida RD8MR3; Kmr	[16]
P. putida RD8MR3PPOR	ppoR::Km of P. putida RD8MR3, Kmr	This study
P. putida WCS358	Wild type; plant growth promoting strain from the rhizosphere of potato roots	[21]
P. putida WCS358PPOR	ppoR::Km of P. putida WCS358, Kmr	This study
P. putida M17	psrA178::Tn5 of P. putida WCS358, Kmr	[23]
P. putida MKO1	rpoS880::Tn5 of P. putida WCS358, Kmr	[22]
P. putida IBE1	gacA400::Tn5 of P. putida WCS358, Kmr	[17]
P. putida IBE2	ppuR1793::Tn5 of P. putida WCS358, Kmr	[17]
P. putida IBE3	rsaL1640::Tn5 of P. putida WCS358, Kmr	[17]
P. putida IBE5	ppuI::Km of P. putida WCS358, Kmr	This study
		
E. coli		
E. coli M15(pRep4)	Derivative of E. coli K-12, containing pREP4 plasmid ensuring the production of high levels of lac repressor protein; Kmr	Qiagen
E. coli Dh5α	F'/endA1 hsdR17 supE44 thi-1 recA1 gyrA relA1 (lacZYA-argF)U169 deoR [80dlac(lacZ)M15recA1]	[26]
A. tumefaciens NTL4 (pZLR4)	A. tumefaciens NT1 derivative carrying a traG::lacZ reporter fusion	[34]
		
Plasmid		
pRK2013	Tra+ Mob+ ColE1 replicon; Kmr	[31]
pMOSBlue i	Cloning vector, Ampr	Amersham-Pharmacia
		
pBBR mcs-5	Broad-host-range vector, Gmr	[29]
pBBRPpoR	pBBR mcs-5 with 749-bp XbaI-KpnI fragment containing ppoR, Gmr	This study
pBluescript KS	Cloning vector, Ampr	Stratagene
pQE30	Expression vector, Ampr	Qiagen
pQEPpoR	721-bp containing ppoR of P. putida KT2440 cloned as SphI-HindIII fragment in pQE30	This study
pMP220	Promoter probe vector, IncP1; Tcr	[28]
pPUI220	ppul promoter cloned in pMP220; Tcr	[17]
pPUR220	ppuR promoter cloned in pMP220; Tcr	[17]
pRSA220	rsaL promoter cloned in pMP220; Tcr	[17]
pPpoR1	ppoR promoter of P. putida RD8MR3 cloned in pMP220; Tcr	This study
pPpoR2	ppoR promoter of P. putida WCS358 cloned in pMP220; Tcr	This study
pMPpprIprom	Promoter of gene pprI cloned in pMP220 vector	[16]
pKNOCK-Km	Conjugative suicide vector; Kmr	[35]
pKNOCKppoR1	Internal fragment of P. putida RD8MR3 ppoR cloned into KpnI-XbaI sites of pKNOCK-Km	This study
pKNOCKppoR2	Internal fragment of P. putida WCS358 ppoR cloned into KpnI-XbaI sites of pKNOCK-Km	This study
pEXPPUIKm	pEXGm containing KpnI-SalI fragment of ppuI::Km	This study
pLAFRppoR	Cosmid clone containing P. putida RD8MR3 ppoR	[16]
pBS1	pBluescript KS carrying the 598-bp pcr product of the P. putida RD8MR3 ppoR promoter region	This study
pBS2	pBluescript KS carrying the 318-bp pcr product of the P. putida WCS358 ppoR promoter region	This study
pBS3	pBluescript KS carrying the 721-bp pcr product of the P. putida KT2440 complete ppoR gene	This study
pBS4	pBluescript KS carrying the 749-bp pcr product of the P. putida WCS358 complete ppoR gene	This study
pBS5	pBluescript KS carrying the HindIII subclone of pLAFRppoR that contains ppoR	This study
pBS6	pBluescript KS carrying the pKNOCK-Km insertion flanking sequences from P. putida WCS358PPOR genomic DNA	This study
pMOS1	pMOSBlue vector carrying 394-bp internal portion of P. putida RD8MR3 ppoR gene	This study
pMOS2	pMOSBlue vector carrying 385-bp internal portion of P. putida WCS358 ppoR gene	This study
pMOS3	pMOSBlue vector carrying pcr product of 358_PpoRf and 4648 degR primers	This study

**Figure 4:**
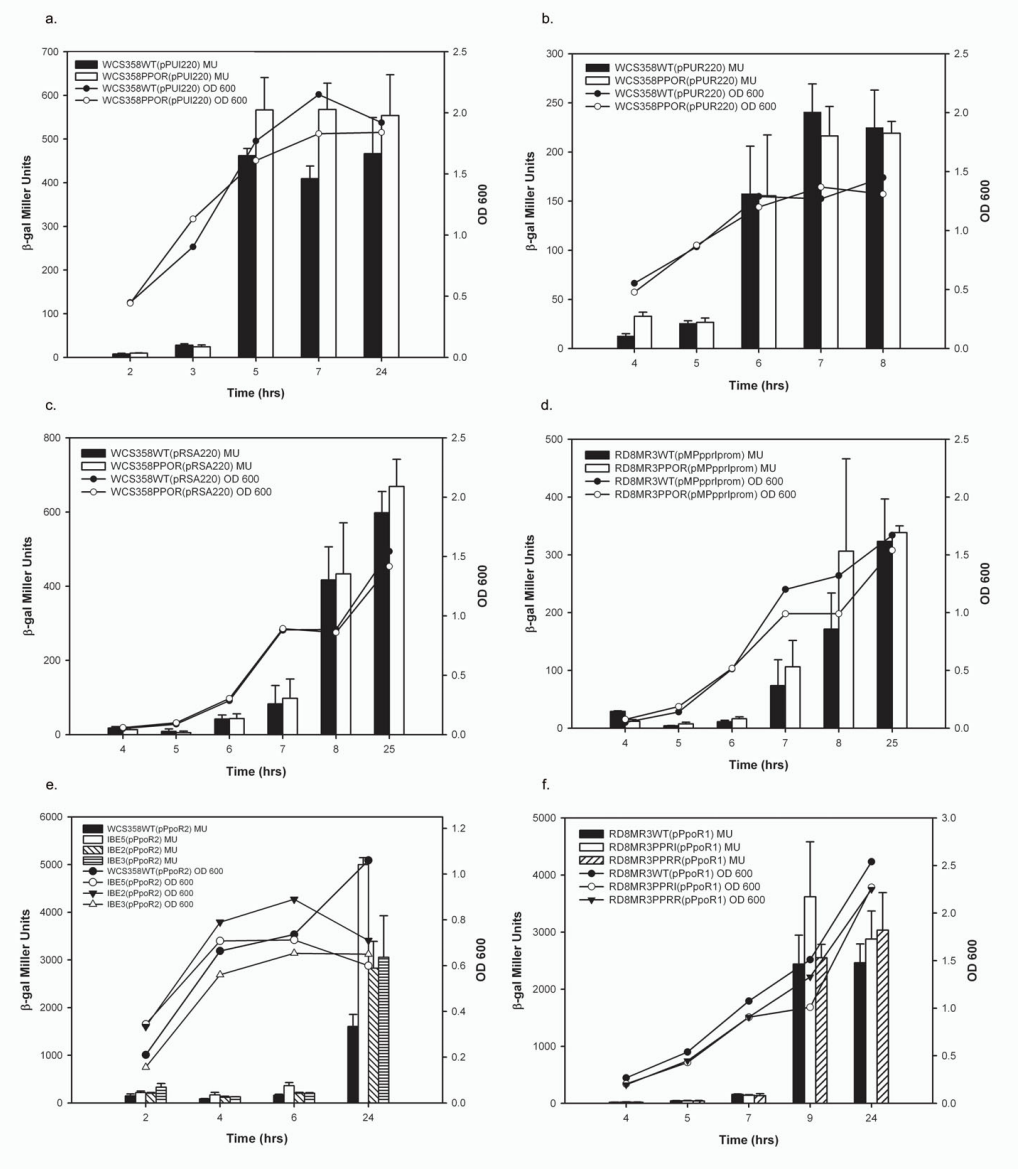
**β-Galactosidase assays showing expression profile of *ppoR *and the QS system genes of *P. putida *WCS358 and RD8MR3**. Bacterial cultures were started with an initial inoculum of 5 × 10^6 ^CFU per ml in 20 ml of minimal medium (M9-Cas) and β-Galactosidase activities were measured at different stages of growth. The growth curves of different mutants and the wild type strain are indicated in each graph. All experiments were performed in triplicate and the mean values of each time point along with standard deviations are shown in each graph. All the graphs were plotted using SigmaPlot version10.0. (**a, b, and c**) *ppuI*, *ppuR *and *rsaL *promoter activities of *P. putida *WCS358 in wild type and WCS358PPOR using plasmids pPUI220, pPUR220 and pRSA220. Paired t-test analysis of *ppuI *promoter activities revealed a significant difference between the mean values of wild type and WCS358PPOR at 7 hours of growth (p value 0.0184; t = 7.268 df = 2) at P < 0.05 significance level. (**d**) *pprI *promoter activity in *P. putida *RD8MR3 wild type and RD8MR3PPOR with the plasmid pMPpprIprom. (**e**) *ppoR *promoter activity in *P. putida *WCS358 wild type, *ppuI *knock-out (IBE5), *ppuR *(IBE2) and *rsaL *(IBE3) mutants with the plasmid pPpoR2. Anova analysis of sample means followed by Dunnett's multiple comparison test revealed that there is a significant difference between the means of wild type and IBE5 at P < 0.05 significance level at 4, 6 and 24 hours growth [F(3,8) = 6.278, F(3,8) = 22.97 and F(3,8) = 16.37 respectively] (**f**) *ppoR *promoter activity in *P. putida *RD8MR3 wild type, *pprI *(RD8MR3PPRI) and *pprR *(RD8MR3PPRR) mutants with the plasmid pPpoR1. β-gal, β-galactosidase; OD600, optical density at 600 nm; MU, Miller Units.

We also determined if PpoR was involved in transcriptional regulation of the QS systems *ppuI/R *of *P. putida *WCS358 and *pprI/R *of *P. putida *RD8MR3. To perform this experiment, *lacZ*-transcriptional promoter probe fusions of *ppuI*, *ppuR *and *rsaL *for *P. putida *WCS358 and *pprI *for *P. putida *RD8MR3 were monitored for expression throughout the growth phase in their respective wild type and *ppoR *mutant strains. For *P. putida *WCS358 QS-related gene promoters, it was observed that *ppuR *and *rsaL *promoters showed comparable expression levels in both wild type and *ppoR *mutant strains at different growth phases (Figures 4b &4c). On the other hand the *ppuI *promoter of *P. putida *WCS358 controlling the AHL synthase exhibited consistently higher expression levels in WCS358PPOR especially in the logarithmic growth phase which was statistically significant (Figure 4a). The *pprI *transcription levels in *P. putida *RD8MR3 were not significantly different from the wild type (Figures 4d)

In order to understand whether *ppoR *expression is under the control of the QS systems of *P. putida *WCS358 and RD8MR3, *ppoR *promoter-*lacZ *reporter fusions of both strains were assayed in their respective QS system mutants and compared with wild type strains at different growth phases. Interestingly, in *P. putida *WCS358, *ppoR *expression shows substantial increase in the IBE5 *ppuI *AHL synthase mutant, indicating a QS system mediated repression of *ppoR *expression (Figure 4e). The *ppoR *promoter levels in this genetic background were not restored to WCS358 wild-type levels by adding exogenously the four AHLs (3-oxo-C6-, 3-oxo-C8-, 3-oxo-C10- and 3-oxo-C12-HSL) produced by WCS358 (data not shown). The reason for this is not known and we cannot exclude that QS is particularly sensitive to growth phase and AHL concentration, thus exogenous addition of AHLs might not necessarily re-establish the conditions present in the wild-type strain.

The expression levels of *ppoR *in *P. putida *WCS358 IBE2 & IBE3 (*ppuR *and *rsaL *mutant respectively), and *P. putida *RD8MR3PPRI and RD8MR3PPRR although higher were not statistically significant (Figures 4e &4f). These results suggest that *ppoR *interaction with the endogenous QS systems in these two *P. putida *strains may not be similar; in strain WCS358 negative regulation (albeit not very strong) of *ppoR *gene expression occurred in response to AHLs via a mechanism which could be independent of the cognate PpuR AHL sensor/regulator.

### *ppoR *expression is growth phase regulated

**Figure 5:**
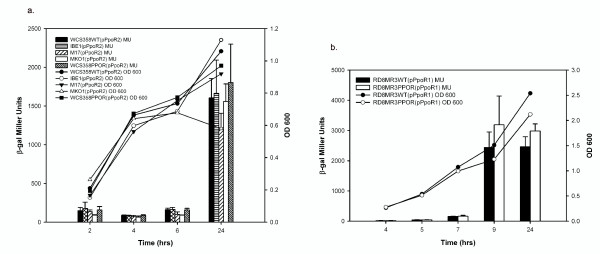
***ppoR *promoter activities in wild type and various mutant strains of *P. putida *WCS358 and RD8MR3**. Bacterial cultures were started with an initial inoculum of 5 × 10^6 ^CFU per ml in 20 ml of minimal medium (M9-Cas) and β-galactosidase activities were measured at different stages of growth. The growth curves of different mutants and the wild type strain are indicated in each graph. All experiments were performed in triplicate and the mean values of each time point along with standard deviations are shown in each graph. All the graphs were plotted using SigmaPlot version10. (**a**) *ppoR *promoter activity in *P. putida *WCS358 wild type, *gacA *(IBE1), *psrA *(M17), *rpoS *(MKO1) and *ppoR *(WCS358PPOR) using plasmid pPpoR2. (**b**) *ppoR *promoter activity in *P. putida *RD8MR3 wild type and *ppoR *(RD8MR3PPOR) mutants using plasmid pPpoR1. β-gal, β-galactosidase; OD600, optical density at 600 nm; MU, Miller Units.

In order to understand if PpoR expression patterns showed any correlation to its role in interacting with the endogenous QS system, *ppoR *expression levels were measured as β-galactosidase activities at different growth phases. Importantly, it was observed for both *P. putida *WCS358 and RD8MR3 that at low cell densities *ppoR *transcription showed minimal expression but was found to increase sharply when the culture enters the logarithmic phase of growth (Figure 5). This pattern of expression level was maintained even in WCS358PPOR and RD8MR3PPOR indicating a lack of regulation by PpoR of its own expression. To find out if *ppoR *expression is under the control of well known growth phase dependent global regulators, its expression level was monitored in *P. putida *WCS358 MKO1 (*rpoS*), M17 (*psrA*) and IBE1 (*gacA*). There was no significant difference in the expression pattern levels of *ppoR *promoter in the three mutants when compared to wild type suggesting that these three global growth-phase regulators were not involved in modulating *ppoR *expression levels (Figure 5). It was therefore concluded that *ppoR *gene expression is stringently growth phase regulated via a yet unidentified regulator.

### Rhizosphere colonization ability of *P. putida *WCS358PPOR & RD8MR3PPOR are not affected

**Figure 6:**
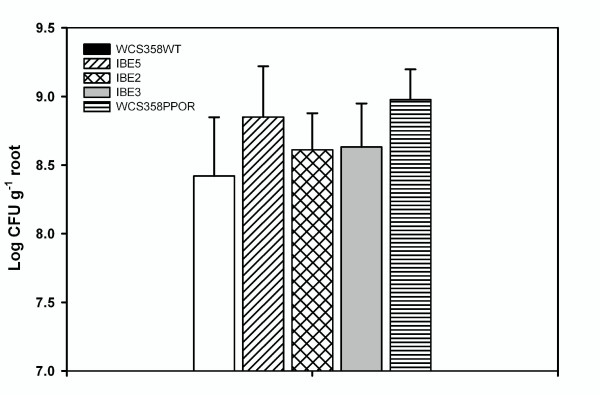
**Root colonization assay of *P. putida *WCS358 wild type and mutants**. Colonization assays were performed as described previously (Steindler et al. 2008). The data presented are from one experiment. Anova analysis in combination with Dunnett's multiple comparison test revealed a significant difference between the mean values of wild type & IBE5 as well as between wild type & WCS358PPOR at P < 0.01 significance level [F(4,45) = 2.870].

Traits involved in surface associated growth of *P. putida *may be regulated by their QS system and possibly also determine their fitness in the rhizosphere [19,20]. Rice root colonization was carried out following the protocol as previously reported [16] with *P. putida *WCS358 wild type, WCS358PPOR and WCS358 QS mutants. Our results revealed that wild type, IBE2 & IBE3 exhibited similar degree of colonization whereas IBE5 & WCS358PPOR were slightly better in colonization of rice roots (Figure 6). One way ANOVA analysis in conjunction with Dunnett's test (P < 0.01) was carried out to confirm that the means of the cell number were significantly different when compared to the wild type strain. Similar experiment with RD8MR3 wild type and RD8MR3PPOR showed that they colonized rice roots to the same extent (data not shown).

### Identification of putative target genes of PpoR by microarrayanalysis

In order to identify target genes directly or indirectly regulated by PpoR, global gene expression comparison was performed of *P. putida *WCS358 wild type with a strain over expressing *ppoR *(PpoR++). Microarray analysis was performed with a single biological sample for each strain with four technical replicates (as mentioned in Methods). Our results revealed that a total of 62 genes show differential expression of more than two fold (P < 0.05) in cultures that were grown in minimal medium (Table 2 and 3). Majority of genes that showed a down regulation of gene expression in the PpoR++ strain were those involved in amino acid catabolism. Genes that showed up regulation of expression in the PpoR++ were those that take part in protein synthesis and sulfur metabolism.

**Table 2:** List of genes showing up regulation of gene expression in *P. putida *WCS358 PpoR^++ ^strain

	Gene name as annotated in *P. putida *KT2440	Function	Fold change
1.	PP0233	Taurine ABC transporter, periplasmic taurine-binding protein	5.016

2.	PP5172	hypothetical protein	4.503

3.	PP0237	sulfonate ABC transporter, periplasmic sulfonate-binding protein SsuA	3.801

4.	PP0236	NADH-dependent FMN reductase	3.751

5.	PP0170	ABC transporter, periplasmic binding protein	3.555

6.	PP0459	50S ribosomal protein L22	3.063

7.	PP0235	antioxidant protein LsfA	3.002

8.	PP0462	50S ribosomal protein L29	2.853

9.	PP0457	50S ribosomal protein L2	2.758

10.	PP0458	30S ribosomal protein S19	2.666

11.	PP5085	malic enzyme	2.665

12.	PP0461	50S ribosomal protein L16	2.631

13.	PP1465	50S ribosomal protein L19	2.626

14.	PP0463	30S ribosomal protein S17	2.602

15.	PP0455	50S ribosomal protein L4	2.592

16.	PP0464	50S ribosomal protein L14	2.563

17.	PP0460	30S ribosomal protein S3	2.455

18.	PP0465	50S ribosomal protein L24	2.431

19.	PP0453	30S ribosomal protein S10	2.426

20.	PP0721	50S ribosomal protein L25	2.334

21.	PP5168	sulfate ABC transporter, ATP-binding protein	2.297

22.	PP0466	50S ribosomal protein L5	2.236

23.	PP0475	50S ribosomal protein L36	2.213

24.	PP1600	outer membrane protein OmpH	2.205

25.	PP1464	tRNA (guanine-N(1)-)-methyltransferase	2.181

26.	PP0454	50S ribosomal protein L3	2.178

27.	PP0689	50S ribosomal protein L27	2.073

28.	PP0470	50S ribosomal protein L18	2.059

**Table 3:** List of genes showing down regulation of gene expression in *P. putida *WCS358 PpoR^++ ^strain

	Gene name as annotated in *P. putida *KT2440	Function	Fold change
1.	PP3433	4-hydroxyphenylpyruvate dioxygenase	18.116

2.	PP2335	citrate synthase	12.097

3.	PP1743	acetate permease	9.109

4.	PP4621	homogentisate 1,2-dioxygenase	7.574

5.	PP1742	hypothetical protein	7.057

6.	PP4064	isovaleryl-CoA dehydrogenase	6.120

7.	PP4065	3-methylcrotonyl-CoA carboxylase, beta subunit, putative	6.042

8.	PP0882	dipeptide ABC transporter, periplasmic dipeptide-binding protein	5.896

9.	PP4402	2-oxoisovalerate dehydrogenase, beta subunit	5.677

10.	PP4864	branched-chain amino acid ABC transporter, ATP-binding protein	5.553

11.	PP4619	maleylacetoacetate isomerase, putative	5.245

12.	PP0545	aldehyde dehydrogenase family protein	5.053

13.	PP2333	transcriptional regulator, GntR family	4.694

14.	PP4866	branched-chain amino acid ABC transporter, permease protein	4.469

15.	PP1140	branched-chain amino acid ABC transporter, permease protein	4.185

16.	PP1000	ornithine carbamoyltransferase	4.006

17.	PP0999	carbamate kinase	3.475

18.	PP0193	hypothetical protein	3.470

19.	PP1001	arginine deiminase	3.335

20.	PP1297	general amino acid ABC transporter, periplasmic binding protein	3.111

21.	PP0764	hypothetical protein	3.100

22.	PP4650	ubiquinol oxidase subunit II, cyanide insensitive	3.073

23.	PP0751	malate:quinone oxidoreductase	2.972

24.	PP0989	glycine cleavage system protein H	2.759

25.	PP0397	hypothetical protein	2.676

26.	PP4975	long-chain acyl-CoA thioester hydrolase family protein	2.601

27.	PP5258	aldehyde dehydrogenase family protein	2.507

28.	PP1690	hypothetical protein	2.469

29.	PP2738	transcriptional regulator, putative	2.463

30.	PP4814	ATP-dependent protease La domain protein	2.338

31.	PP3122	CoA-transferase, subunit A, putative	2.176

32.	PP4194	citrate synthase	2.162

33.	PP0684	peptidyl-prolyl cis-trans isomerase, FKBP-type	2.077

34.	PP5319	hypothetical protein	2.013

**Figure 7:**
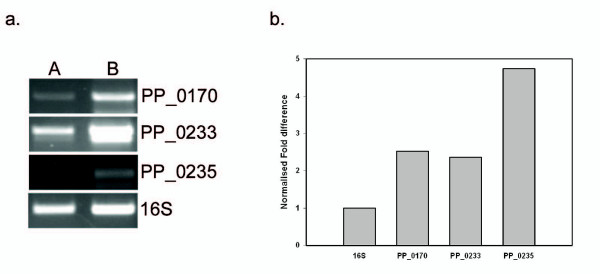
**RT-PCR analysis to validate expression of genes in *P. putida *WCS358**. Total RNA isolations were carried out from bacterial cultures grown in minimal M9 medium using Ribopure RNA isolation kit (Ambion) and DNase treatment was carried out. cDNA synthesis was done using AMV Reverse Transcriptase (Promega) and second strand synthesis performed using Go Taq Flexi polymerase (Promega). RT-PCR analysis was performed with RNA obtained from two independent isolations and the figure shows results of one such experiment. (**a**) Agarose gel showing RT-PCR products for the genes PP_0170, PP_0233 and PP_0235. RT_PCR for 16S rRNA was carried out from the same RNA samples as control to ensure that equal amounts of RNA were taken. **A**. RT-PCR on RNA sample from *P. putida *WCS358 containing pBBR vector alone and **B**. RT-PCR on RNA sample from *P. putida *WCS358 containing pBBRPpoR. (**b**) Graph showing normalized fold difference of genes when compared to 16S rRNA expression levels. The gel image containing bands was analyzed by the ImageJ software and the bars indicate the fold increase in the intensity of the bands in PpoR++ strain (*P. putida *WCS358 containing pBBRPpoR) when compared to wild type (*P. putida *WCS358 containing pBBR vector alone).

In order to validate the differential expression of genes observed in the microarray experiment, semi quantitative RT PCR analysis of three genes PP_0170, PP_0233 and PP_0235, was performed as they were among genes that showed maximum up-regulation in PpoR++ strains when compared to wild type. Briefly, PP_0170 codes for a putative ABC transporter periplasmic binding protein (3.55 fold up regulation in PpoR++ strain), PP_0233, designated as *tauA*, encodes a putative taurine ABC transporter periplasmic binding protein (5 fold up regulation in PpoR++ strain) and PP_0235, named *lsfA*, codes for a putative peroxidase (3 fold up regulation in PpoR++ strain). RT PCR analysis with two independent RNA isolations shows more than two fold increases in expression of these genes in PpoR++ strain when compared to wild type and is in agreement with the results obtained in microarray (Figure 7). As these genes take part in inorganic ion utilization and oxidative stress, it is possible that PpoR might play a functional role in these processes.

## Conclusion

The roles of solo QS LuxR proteins in inter-species as well inter-kingdom signaling are just beginning to be understood with a few recent studies on these proteins in non-AHL producing bacteria. The extent of the functional participation/interaction of these proteins in QS in AHL producing bacteria also differs depending on the strain. We have characterized PpoR, a solo LuxR homolog present in both AHL and non-AHL producing bacteria; its conservation indicates a significant role for this protein of *P. putida*. In fact most strains of *P. putida *do not harbor an AHL quorum sensing system, however they possess PpoR indicating that it is likely to be part of the core genome of this species. We have shown that PpoR binds AHLs and that it is highly conserved in *P. putida*; and this in our view represents the important novel finding of our study., In addition we believe that we are in a position to conclude that the results obtained using our strain represent what occurs in *P. putida *strains (including the ones which only have PpoR and do not contain a complete AHL QS system). Future studies will be directed towards understanding the regulation of target genes in response to exogenous AHLs in certain *P. putida *strains and also possibly endogenous AHLs in strains which harbor an AHL QS system.

## Methods

### Bacterial strains, plasmids and media

All strains, plasmids and primers used in this study are listed in Tables 1 and 4. *P. putida *[21-24] and *E. coli *strains were grown in Luria-Bertani (LB; [25]) medium at 30 and 37°C respectively. *P. putida *strains were also grown in M9 minimal medium [26] supplemented with 0.3% casamino acids (M9-Cas) at 30°C. *Agrobacterium tumefaciens *NTL4 (pZLR4) was grown in AB medium [27] at 28°C. Antibiotics when required were supplemented at the following concentrations: ampicillin, 100 μg/ml; kanamycin, 100 μg/ml (*Pseudomonas*) or 50 μg/ml (*E. coli*); nalidixic acid, 25 μg/ml; tetracycline, 10 μg/ml (*E. coli*) or 40 μg/ml (*Pseudomonas*); and gentamicin, 10 μg/ml (*E. coli*) or 40 μg/ml (*Pseudomonas*). Transcriptional fusion constructs for *ppoR *promoter in pMP220 [28] were made as follows: a 598-bp fragment containing the *ppoR *promoter region was amplified from *P. putida *RD8MR3 genomic DNA with the primers 16orpF and 16orpR using Vent DNA polymerase (New England Biolabs) following supplier's instructions, cloned in pBluescript (Stratagene) yielding pBS1 and verified by DNA sequencing (Macrogen Inc., Korea). The *ppoR *promoter was removed as a KpnI-XbaI fragment from pBS1 and cloned in pMP220 yielding pPpoR1. Similarly, a 318-bp fragment was amplified from *P. putida *WCS358 genomic DNA using primers 358orpromF and 358orpromR and cloned in pBluescript yielding pBS2. The *ppoR *promoter was removed as KpnI-XbaI fragment from pBS2 and cloned in pMP220 yielding pPpoR2. To clone *ppoR *gene in pQE30, a 721-bp fragment containing the entire *ppoR *gene of *P. putida *KT2440 was amplified using primers KT_PpoRf and 4647R1 and cloned in pBluescript yielding pBS3. The *ppoR *gene was removed as SphI-HindIII fragment and cloned in pQE30 in the correct reading frame yielding pQEPpoR. To clone *ppoR *in pBBR [29], the 749-bp fragment containing the entire *ppoR *gene was amplified using *P. putida *WCS358 genomic DNA as the template using primers 358_PpoRf and 358_PpoRr and cloned in pBluescript yielding pBS4. *ppoR *gene was excised from pBS4 using XbaI-KpnI and cloned into pBBR mcs-*5 *yielding pBBRPpoR.

**Table 4:** Primers used in this study

Primer	Sequence	Reference or source
16F	CGACGCAAACGCCACGGT	This study
16R	TGCTCATCTTGGGCTGCG	This study
putidadegF	GCCTGCGCCACGG(AGCT)TGGAC(AGCT)CA	This study
putidadegR	GATCACGGAGCGGATGTG(AG)AA(AG)TT(AGCT)AC	This study
16orpF	CATCAGCCTTGGTCACGCC	This study
16orpR	GCGTCCTGCTTGTAGAACTC	This study
358orpromF	GCACAGTGTCGCGCAAAGC	This study
358orpromR	ATGTTCAGCCCCAAGTGTTC	This study
358_PpoRf	CCATCCATAGGAGCGTTACG	This study
358_PpoRr	GGTAGTCGCAGGGGTGGCTA	This study
KTPpoRf	AGGCATGCCTTCACTGGAAACCCGAG	This study
PP4647R1	GGAAGCTTTCAGATCCAGCCACGCAA	This study
PP4647F	GGAATTCGAAACCCGAGTATCTTCGTG	This study
PP4647R	GGGTACCGGTGATCACGCTGCGGATATG	This study
4648degR	CCCTGCAGGCGGATCAR(CT)TG(CT)TC(CT)TC	This study
358PpoRintF	GCAGCACAATGAAAGCCAAG	This study
358PpoRintR	GTGACAGCGACAGGATGGAG	This study
KT_16For	AGTTGGTGGGGTAATGGCTC	This study
KT_16Rev	TGTCAGTATCAGTCCAGGTG	This study
PP0170For	ATCGTCTACCTGCTGCTGAA	This study
PP0170Rev	AGCAAACAGCAAGGTCGGCG	This study
PP0233For	AAACCTTCCTCATCGCCACC	This study
PP0233Rev	TCCTTGCGGTAGTCGGCGTA	This study
PP0235For	GCTGAAGGACGAGTTTGCCA	This study
PP0235Rev	ATCACCACTTCGTCGCCGTC	This study

### Recombinant DNA techniques

DNA manipulations, including digestion with restriction enzymes, agarose gel electrophoresis, purification of DNA fragments, ligation with T4 ligase, end filling with the Klenow enzyme, hybridization, radioactive labeling by random priming, and transformation of *E. coli*, were performed as described previously [26]. Southern hybridizations were performed by using N+Hybond membranes (Amersham Biosciences); plasmids were purified by using Jet star columns (Genomed GmbH, Löhne, Germany); and total DNA from *Pseudomonas *was isolated by Sarkosyl-pronase lysis as described previously [30]. Triparental matings between *E. coli *and *P. putida *were carried out with the helper strain *E. coli *DH5α (pRK2013) [31].

### AHL binding assay

50 ml cultures of *E. coli *M15(pRep4) strains containing either pQE30 or pQEPpoR were grown at 37°C to an initial optical density at 600 nm of 0.1 (OD 600). 5 ml aliquots of these cultures were supplemented with different types of AHLs at 1 μM concentration and grown at 30°C until OD 600 of 0.6. Protein expression was induced by IPTG (1 μM final concentration) and the cultures were incubated further with shaking for 3.5 hours. OD 600 was measured and equal numbers of cells were processed for all samples as follows; the cell pellets were washed three times with PBS before finally resuspending in equal volume of PBS. These cell suspensions were extracted once with the same culture volume of ethyl acetate-0.1% acetic acid. The extracts were then dried, resuspended in ethyl acetate and analyzed by thin layer chromatography (TLC) performed with C_18 _reverse-phase chromatography plates (Shaw *et al.*, 1997). The AHL molecules were detected by overlaying the TLC plate with a thin layer of AB top agar seeded with *A. tumefaciens *NTL4 (pZLR4) in presence of 100 μg/ml X-gal.

### AHL extraction, visualization and quantification

AHLs were purified from bacterial strains and analyzed either on a C_18 _reverse-phase chromatography TLC plates as previously described (Shaw *et al.*, 1997) or quantified as previously described Rampioni et al [32] for 3-oxo-C12-HSL or by Steindler et al., [16] for 3-oxo-C6-HSL. For visualization on TLC, the extracts were placed on a TLC plate and AHLs were separated as previously described [33] and the plate was then overlaid with a thin layer AB top agar seeded with *A. tumefaciens *NTL4 (pZLR4) [34] in presence of 100 μg/ml X-gal, as described previously [33].

### Cloning of the *ppoR *gene of *P. putida *RD8MR3 and WCS358, generation of *ppoR *mutants in both strains and of a *ppuI *mutant in WCS358

The *P. putida *RD8MR3 *ppoR *gene was cloned as follows; *P. putida *KT2440 partial *ppoR *gene was amplified using primers PP_4647F and PP_4647R and used as probe to screen a cosmid library of *P. putida *RD8MR3 [16] by colony hybridization. Cosmid pLAFRppoR was identified, *ppoR *gene localized to a 4.5-kb HindIII fragment and cloned in pBluescript to yield pBS5 which was sequenced using vector specific primers and by primer walking to obtain 1735-bp containing RD8MR3 *ppoR*. To generate a *ppoR *mutant in strain RD8MR3, we constructed pKNOCKppoR1 as follows; a 394-bp internal fragment of *P. putida *RD8MR3 *ppoR *gene was amplified by PCR using primers 16F and 16R and cloned in pMOSblue yielding pMOS1. *ppoR *internal fragment was excised from pMOS1 using XbaI-KpnI and cloned into pKNOCK-Km [35] to yield pKNOCKppoR1. pKNOCKppoR1 was used as suicide vector to create knockout mutants of *ppoR *by homologous recombination in *P. putida *RD8MR3 designated RD8MR3PPOR. The fidelity of the marker exchange events was confirmed by Southern analysis of mutants.

In order to generate a *ppoR *mutant in strain WCS358, we constructed pKNOCKppoR2 as follows; a 385-bp internal fragment of *P. putida *WCS358 *ppoR *gene was amplified by PCR using degenerate primers putidadegF and putidadegR and cloned in pMOSblue yielding pMOS2. *ppoR *internal fragment was excised from pMOS2 using XbaI-KpnI and cloned into pKNOCK-Km generating pKNOCKppoR2. pKNOCKppoR2 was then used as a suicide vector to create knockout mutants of *ppoR *by homologous recombination in WCS358 designated WCS358PPOR. The fidelity of the marker exchange events was confirmed by Southern analysis of mutants. In order to clone the *ppoR *gene from *P. putida *WCS358, the genomic DNA of WCS358PPOR (generated as mentioned above) was digested with an enzyme flanking vector insertion on one side and cloned into pBluescript to yield pBS6. Sequencing of this clone using vector specific primers yielded an 1148-bp sequence covering the promoter and the first 570-bp of *ppoR*. The last 135-bp of the *ppoR *gene was obtained by amplification of this region from *P. putida *WCS358 wild type using primers 358_PpoRf and 4648degR (a degenerate primer based on available *P. putida *sequences of the downstream gene PP_4648), cloning in pMOS to yield pGEM3 and sequencing of pMOS3 with vector specific primers.

In order to generate a *ppuI *AHL synthase mutant in WCS358 we introduced a Km resistance cassette removed as a HincII fragment into the BsaAI site of *ppuI *harbored in pBQS1 generating pQS1:Km. ppuI::Km was then cloned into pEXGm as a KpnI-SalI fragment generating pEXPPUIKm. This latter plasmid was used in generating a *ppuI *knock mutant, designated *P. putida *IBE5, via homologous recombination and selection as previously described [36].

### Reporter gene fusion assay and root colonization

β-galactosidase activities were determined during growth in M9-Cas essentially as described by Miller [25] with the modifications of Stachel et al[37]. All experiments were performed in triplicate, and the mean values are given. Statistical significance of the values were calculated by paired t-test (to compare two sample mean values; P < 0.05) or one way anova in combination with Dunnett's test (to compare multiple sample mean values; P < 0.05). β-galactosidase activities were determined at various times after a 20-ml M9-Cas culture was started with an initial inoculum of 5 × 10^6 ^CFU. Root colonization assays were performed exactly as described by Steindler et al [16].

### Total RNA isolation

An overnight culture of *P*. *putida *WCS358 strains carrying pBBR mcs-*5 *or pBBRPpoR grown in M9-Cas was used to obtain an initial OD 600 of 0.1. The cultures were incubated at 30°C on a rotary shaker at 180 rpm until they reached an OD 600 of approximately 1.2. RNA isolation was carried out from 2 × 10^9 ^cells using Ribopure™-bacteria RNA isolation kit (Ambion Inc., Austin, USA) as per manufactures's instructions. DNase treatment of RNA was done at 37°C for 1 hour (Ambion), if necessary twice and RNA purified. The purity of RNA was assessed by performing a PCR on a fixed quantity of total RNA (250 ng) with GoTaq polymerase (Promega) using genomic DNA as control with 358PpoRintF and 358PpoRintR primers specific for *P. putida *WCS358 *ppoR*. The RNA quality was assessed by spectrophotometric measurement at 260 nm and 280 nm and its intact nature verified by visualizing RNA samples on an agarose gel.

### Microarray analysis

A customized high-density oligonucleotide whole genome expression array (NimbleGen Systems Inc., Madison, WI) was designed for *P. putida *KT2440 using the genome sequence and open reading frame (ORF) predictions available from GenBank accession number NC_002947. The 6,181,863-bp chromosome of KT2440 contains 5,350 predicted ORFs and 96 RNAs. 60-mer probes paired with perfect-match (PM) oligonucleotides and their corresponding mismatch oligonucleotides were selected for 5350/5350 sequences with the median number of probes/sequence being 18. Each probe was replicated 4 times on the chip representing a technical replicate. The cDNA synthesis, hybridization, and scanning were performed by NimbleGen Systems Inc. Microarray data analysis was performed using the robust multiarray average method [38] based on the log_2 _values of the absolute signal intensities for PM probes only. Student's *t *test for each PM probe and each technical replicate, followed by the Bonferroni correction, was used to identify genes showing differential expression patterns (*P *< 0.05). The data presented are the results from one experiment.

### Semi quantitative RT PCR and analysis

Reverse transcription was performed in a 20-μl reaction mixture containing 2 μg of total RNA, 100 ng of random primers/μg of RNA and 5 U of AMV reverse Transcriptase (Promega, Madison, WI) following manufacturer's instructions. After denaturing RNA and random primers at 65°C for 3 min, the remaining reagents were added and the mixture incubated at 25°C for 10 min, 42°C for 90 min and held at 70°C for 10 min to inactivate the enzymes. The KT_16For and KT_16Rev primers were used to measure the transcription of 16S rRNA. Second strand synthesis was performed using Go Taq Flexi polymerase (Promega) using 1 μl of cDNA reaction as template; for 16S rRNA, 1 μl of 1:100 diluted cDNA reaction was used. The number of PCR cycles to be performed for each gene was standardized so that the product amplification is in the linear range and proportional to the amount of input sample. 10 μl of the PCR reaction was analyzed by agarose gel electrophoresis. The intensity of the bands obtained were measured and normalized to that of 16S rRNA using the ImageJ software [39] to obtain the fold difference. Each gene was validated twice by RT PCR analysis of RNA samples from two independent isolations.

### Nucleotide sequence accession numbers

All DNA sequences were performed at Macrogen http://www.macrogen.com and the nucleotide sequences were deposited in GenBank/EMBL/DDBJ; *ppoR *gene of *P. putida *RD8MR3 is given under accession number FM992078 whereas the *ppoR *gene of *P. putida *WCS358 is given under accession number FM992077.

## Authors' contributions

SS carried out all the experimental studies and participated in experimental design and drafting the manuscript. VV designed, coordinated the study and drafted the manuscript. Both authors read and approved the final manuscript.
